# Analysis of a Lateral Grain Boundary for Reducing Performance Variations in Poly-Si 1T-DRAM

**DOI:** 10.3390/mi11110952

**Published:** 2020-10-22

**Authors:** Songyi Yoo, Wookyung Sun, Hyungsoon Shin

**Affiliations:** 1 Department of Electronic and Electrical Engineering, Ewha Womans University, Seoul 03760, Korea; dbthddl0219@ewhain.net; 2 Smart Factory Multidisciplinary Program, Ewha Womans University, Seoul 03760, Korea; 3 Department of Electrical and Computer Engineering, Seoul National University, Seoul 08826, Korea

**Keywords:** capacitorless one-transistor dynamic random-access memory, 1T-DRAM, polysilicon, grain boundary, lateral grain boundary (GB), GB location

## Abstract

A capacitorless one-transistor dynamic random-access memory device that uses a poly-silicon body (poly-Si 1T-DRAM) has been suggested to overcome the scaling limit of conventional one-transistor one-capacitor dynamic random-access memory (1T-1C DRAM). A poly-Si 1T-DRAM cell operates as a memory by utilizing the charge trapped at the grain boundaries (GBs) of its poly-Si body; vertical GBs are formed randomly during fabrication. This paper describes technology computer aided design (TCAD) device simulations performed to investigate the sensing margin and retention time of poly-Si 1T-DRAM as a function of its lateral GB location. The results show that the memory’s operating mechanism changes with the GB’s lateral location because of a corresponding change in the number of trapped electrons or holes. We determined the optimum lateral GB location for the best memory performance by considering both the sensing margin and retention time. We also performed simulations to analyze the effect of a lateral GB on the operation of a poly-Si 1T-DRAM that has a vertical GB. The memory performance of devices without a lateral GB significantly deteriorates when a vertical GB is located near the source or drain junction, while devices with a lateral GB have little change in memory characteristics with different vertical GB locations. This means that poly-Si 1T-DRAM devices with a lateral GB can operate reliably without any memory performance degradation from randomly determined vertical GB locations.

## 1. Introduction

Conventional one-transistor one-capacitor dynamic random-access memory (1T-1C DRAM) cells are severely limited in integration density because it is difficult to miniaturize their capacitors, so capacitorless 1T-DRAM has attracted attention as a promising alternative [1,2,3,4,5,6,7,8,9,10,11,12,13,14]. 1T-DRAM devices composed of one silicon-on-insulator (SOI) transistor have different operating mechanisms depending on their body material [15]. A 1T-DRAM device with a silicon body differentiates its state using current differences in the number of holes stored in its floating body (FB). Although a silicon body 1T-DRAM device can have a small cell size of 4F^2^, it cannot operate as a memory in a fully depleted-SOI (FD-SOI) structure that lacks a FB hole storage region. This means that short channel silicon 1T-DRAM devices with small depletion regions have significantly deteriorated memory performance [15].

In recent years, 1T-DRAM devices with poly-Si bodies have been studied to overcome the limitations of silicon 1T-DRAM [15,16,17,18,19,20,21,22,23,24,25,26]. Since the poly-Si devices use grain boundaries (GBs) instead of a FB as a charge storage region, they can perform memory operations in a FD-SOI structure [15]. Polysilicon has GBs due to its multiple single-crystalline silicon composition; a poly-Si 1T-DRAM device distinguishes its data by using the charge trapping characteristic of GBs. Also, poly-Si 1T-DRAM devices enable a three-dimensional stack architecture with high integration density and they are cost-effective to fabricate because they can be manufactured by annealing deposited amorphous silicon. In several studies, the fabrication of a poly-Si 1T-DRAM cell and its electrical properties were investigated [24,25]; the authors verified that a poly-Si 1T-DRAM cell operates stably as a memory by analyzing the transient characteristics of successfully manufactured memory devices.

In our previous study, we proposed a method of optimizing the body thickness and bias conditions to simultaneously consider the memory performance and the short channel effects (SCEs) of a short-channel poly-Si 1T-DRAM cell [18]; we confirmed that thick body devices with a larger GB area have better memory performance than thin body devices because they have a larger amount of trapped charge. Also, in other studies, we verified that the sensing margin and retention time of a poly-Si 1T-DRAM cell are significantly affected by the number and location of the vertical GBs randomly determined during fabrication [15,17]. The memory performance of a poly-Si device in which a single GB is located near the source and drain is significantly reduced; the device’s sensing margin is inversely proportional to the number of GBs in its body. This makes it necessary to study other optimization methods to improve the memory stability of poly-Si 1T-DRAM cells that have randomly distributed vertical GBs.

In contrast to the difficulty of controlling vertical GB positioning, the distribution of lateral directional GBs can be easily controlled by using multiple discrete depositions during polysilicon fabrication, enabling the formation of almost uniform GBs in the lateral direction [27,28,29]. This paper reports on the simulated memory performance of poly-Si 1T-DRAM devices as a function of their lateral GB’s location and their lateral GB’s presence or absence for varied vertical GB locations. The remainder of this paper is organized as follows: In Section 2, the cross-sections and parameters of simulated devices are described and the biasing and time conditions for transient simulation are presented. In Section 3.1, the sensing margin and retention time dependence on the location of a lateral GB is analyzed and in Section 3.2, we compare memory performance changes for varied vertical GB locations in devices with and without a lateral GB. The conclusions are presented in Section 4.

## 2. Materials and Methods

**Figure 1:**
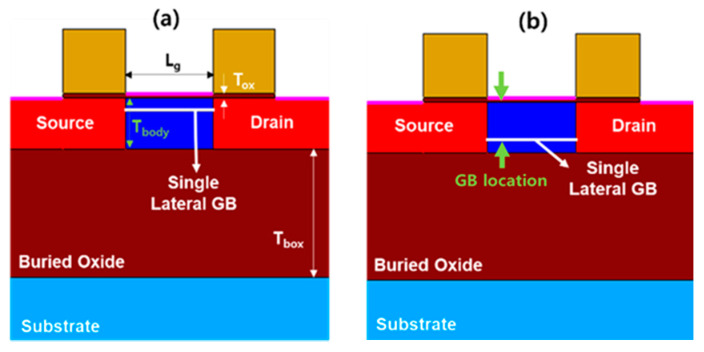
The simulated structure of poly-Si one-transistor one-capacitor dynamic random-access memory (1T-DRAM) cell with two lateral grain boundary (GB) locations. The lateral GB distances from the SiO_2_/poly-Si interface are depicted as (**a**) 10 nm and (**b**) 30 nm.

Simulations were performed with the Sentaurus technology computer aided design (TCAD) tool to investigate the memory performance of a poly-Si 1T-DRAM device as a function of a lateral GB’s location. Figure 1a,b show two of the simulated poly-Si 1T-DRAM devices with different locations of a single lateral GB. The structure is a single gate FD-SOI transistor. We specified the lateral GB’s location by its distance from the SiO_2_/poly-Si interface.

For example, Figure 1a shows the lateral GB at 10 nm and Figure 1b shows it at 30 nm. We simulated the sensing margins and retention times for GB locations of 0 to 40 nm in 2 nm increments. The width of the simulated device is set to 1 um which is a default value for TCAD device simulation. The parameter values other than the lateral GB location for all devices are the same and are shown in Table 1.

Table 2 shows the biasing and timing conditions for transient simulation. The same parameter values were applied to all devices regardless of their lateral GB location. In the Write “1” operation, excess holes are generated by band-to-band tunneling for 500 ns; in the Write “0” operation, electrons are supplied due to negative drain biasing for 150 ns. In the Read operation, the voltages of the inversion conditions was used. A low drain voltage for non-destructive operation and a gate voltage that maximized the device sensing margin were applied for 10 ns. In the Hold operation, 0 V was applied to both drain and gate to maintain the data and the operation time was set as a variable parameter to investigate the current change dependence on the hold time.

## 3. Results and Discussion

### 3.1. Transient Characteristics Depending on the Lateral GB Location

**Figure 2:**
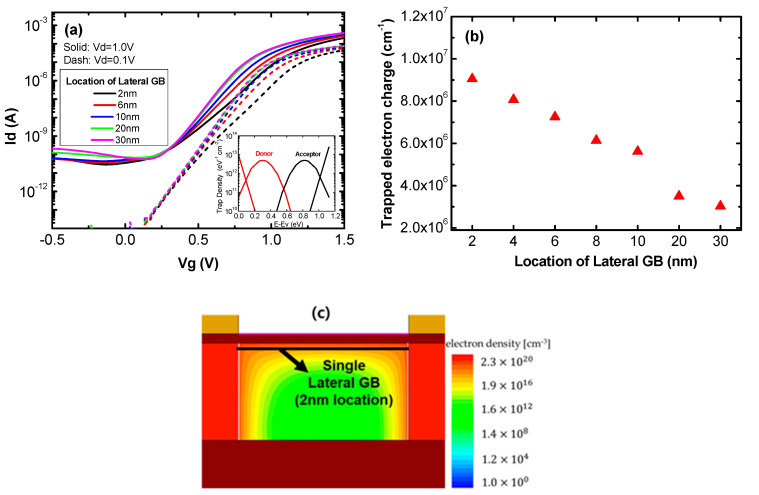
(**a**) Transfer curves of poly-Si 1T-DRAM cells with varied lateral GB locations (inset, the trap densities used in the simulations); (**b**) trapped electron charge according to the location of a single lateral GB; and (**c**) the electron density contour of a device with a lateral GB at 2 nm.

The inset in Figure 2a shows trap densities according to band energy. We set the simulation trap parameter based on recent studies [15,16,17,18]. In the recent research of Reference [16], the investigators verified that the transient drain current is a function of the trap densities; donor type traps have little influence on the drain current while acceptor type trap densities are inversely proportional to the drain current. The red and black lines represent donor and acceptor trap densities, respectively. The trap densities have an exponential distribution in the tails of the conduction and valance bands; they have a Gaussian distribution near the mid-gap. The donor traps are positively charged when electrons are emitted and the acceptor traps are negatively charged when electrons are captured. Figure 2a shows the transfer curves of poly-Si 1T-DRAM devices with varied lateral GB locations. The figure shows that the on current increases as the lateral GB location moves away from the electron channel due to the amount of trapped electron charge. Figure 2b shows that the trapped electron charge is inversely proportional to the lateral GB location. Trapped electron charge reduces the number of free electrons, thus impeding the flow of current and increasing the threshold voltage. Figure 2c shows the electron density of a 2 nm lateral GB location device when the drain and gate voltages are 0.1 V and 1.5 V, respectively. The figure shows that inversion current is concentrated in the 40 Å thick channel, significantly degrading the on current of this device with its lateral GB located inside the channel.

**Figure 3:**
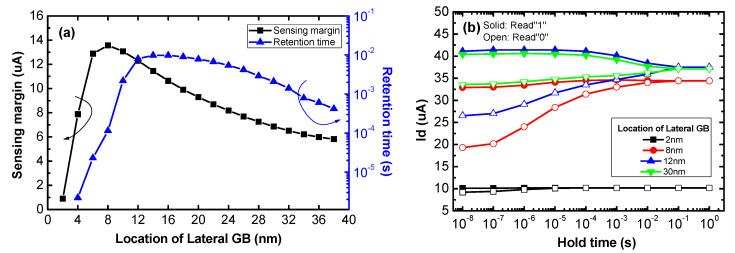
(**a**) The sensing margin and retention time according to the location of the lateral GB and (**b**) the transient characteristics of poly-Si 1T-DRAM devices with varied lateral GB locations.

Figure 3a shows the sensing margins and retention times of poly-Si 1T-DRAM cells with varied lateral GB locations. The sensing margin is defined as the read current difference between the “1” and “0” states with 10 ns of hold time. Retention time is defined as the hold time when the current difference reaches 3 uA, the minimum value for sensing [30]. Devices with lateral GBs located from 2 nm to 6 nm tend to have sensing margins and retention times that increases rapidly as the GB location moves away from the channel. The devices with locations from 8 nm to 14 nm have a trade-off relationship between sensing margin and retention, thus sensing margin decreases while retention time increases. For GB locations larger than 16 nm, sensing margin and retention time both decrease in inverse proportion to the location. The amount of charge trapped in the lateral GB depends on the lateral GB location and this changes the sensing margin and retention time.

Figure 3b shows the transient characteristics of 2 nm, 8 nm, 12 nm and 30 nm lateral GB location devices; these representative locations can show the trends of memory performance. The solid and open symbols indicate the read current after Write “1” and Write “0” operations, respectively. The *x*-axis represents the Hold operation time after writing. As shown in Figure 3b, each device has different retention characteristics. In the 2 nm lateral GB location device, both the Read “1” and Read “0” currents are significantly degraded compared to the other devices. In the 8 nm device, the Read“1” current is almost constant while the Read “0” current increases with hold time and in the 12 nm device, both the Read “1” and Read “0” currents change with the hold time. The Read “0” current hardly changes while the Read “1” current decreases with hold time in the 30 nm lateral GB location device.

**Figure 4:**
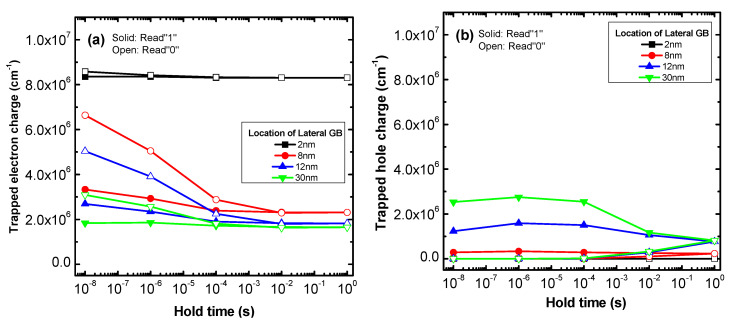
Trapped (**a**) electron and (**b**) hole charge of poly-Si 1T-DRAM devices with varied lateral GB locations according to the hold time in the Read “1” and Read “0” operations.

Figure 4a,b show the trapped electron charge and trapped hole charge of 4 memory devices having the lateral GB locations in Figure 3b. As seen in Figure 4a, the trapped electron charge for Read “0” is larger than Read “1” due to the Write “0” operation electron supply. Also, the trapped electron charge decreases as the lateral GB location increases because the Read operation is performed under the strong inversion conditions forming the electron channel and the Write “0” operation is performed using negative drain bias. Therefore, the trapped electron charge in the Read “0” state increases when the lateral GB is adjacent to the electron channel and drain contact. These trapped electron charge trends are represented in the change of the Read “0” current shown in Figure 3b.

The device with a 2 nm lateral GB location has a significantly larger trapped electron charge in both the Read “0” and Read “1” operations than other devices, thus this device has degraded on current regardless of the states and hold times. Also, the trapped electron charge of the 8 nm, 14 nm lateral GB location devices decreases with hold time due to electron de-trapping which causes an increase in Read “0” current. However, the device with a lateral GB at 30 nm has difficulty trapping electron charge in Read “0” because its GB location is far from the channel and drain contact. This means that the trapped electron charge of the 30 nm device has little influence on its retention characteristics, thus the “0” current hardly changes with hold time.

In Figure 4b, the trapped hole charge in Read “1” is larger than in Read “0” due to the Write “1” operation that generates excess holes. Also, the trapped hole charge is proportional to the lateral GB location because the hole charge trapped in the GB near the channel recombines with the electrons concentrated in the inversion channel. These trapped hole charge trends indicate the change in the Read “1” current of the retention characteristics. The 2 nm and 8 nm lateral GB devices with locations that are relatively close to the electron channel have less trapped hole charge even when the hold time is small and the charge does not change with hold time compared with the 12 nm and 30 nm lateral GB devices. Therefore, the Read “1” current of the 12 nm and 30 nm devices decreases with hold time due to hole de-trapping while that of the 2 nm and 8 nm devices is almost constant. Another remarkable result illustrated in Figure 4a,b is that there is less trapped hole charge during the Read “1” operation than trapped electron charge in the Read “0” operation regardless of the lateral GB location. This is because the simulated device is a FD-SOI structure, so excess hole charge is easily depleted under strong inversion conditions.

The trade-off relationship between the sensing margin and retention time in the 8–14 nm lateral GB location devices is caused by hole charge behavior. For the devices with higher than 8 nm lateral GB location, the sensing margin decreases due to the reduction of the trapped electron charge. Although the trapped hole charge increases, the reduction of trapped electron charge has a greater effect on the sensing margin because the trapped hole charge is smaller than the electron charge in a FD-SOI device. However, in the 8–14 nm devices, retention time increases temporarily because hole de-trapping is slower than electron de-trapping due to its lower mobility. For GB locations larger than 14 nm, retention time tends to decrease as sensing margin decreases without a trade-off trend.

An optimized lateral GB location that considers both the sensing margin and retention time should be determined. Therefore, for this paper, we chose 12 nm as the optimized lateral GB location because the sensing margin and retention time are each near their respective maximum at that location.

### 3.2. Transient Characteristics Depending on the Vertical GB Location

**Figure 5:**
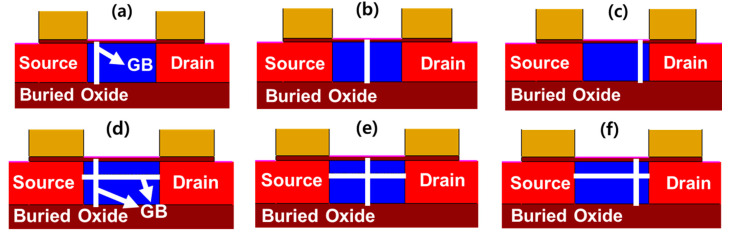
The simulated structure of poly-Si 1T-DRAM devices with different GB distributions. In (**a**–**c**), the devices have no lateral GB and in (**d**–**f**), they have a lateral GB at 12 nm. Vertical GBs are located near the source in (**a**) and (**d**); at the channel center in (**b**) and (**e**); and near the drain in (**c**) and (**f**).

Vertical GBs are randomly formed during poly-Si 1T-DRAM device fabrication and the devices have differing memory performance depending on the distribution of these GBs. Consequently, we investigated the effect of a lateral GB on memory performance by simulating devices having varied vertical GB positions with and without a lateral GB. Figure 5a–f show cross-sections of some simulated poly-Si 1T-DRAM devices that have different locations of a single vertical GB. Figure 5a–c have no lateral GB while Figure 5d–f have a single lateral GB. Figure 3a,b depict the sensing margin and retention time for the optimized lateral GB position at 12 nm; this position was used for the simulations. As the channel length decreases, the number of GBs formed in a poly-Si body is limited, thus a single vertical GB is simulated. In this paper, we named the devices in Figure 5a–d as Vertical_Source, Figure 5b,e as Vertical_Center and Figure 5c,f as Vertical_Drain to correspond to the vertical GB location. In the transient simulation, the gate read voltage that maximizes the sensing margin of a memory cell is 1.2 V in Figure 5a–c and 1.3 V in Figure 5d,f. Other device parameters and simulation conditions are the same as those used in Figure 1a,b.

**Figure 6:**
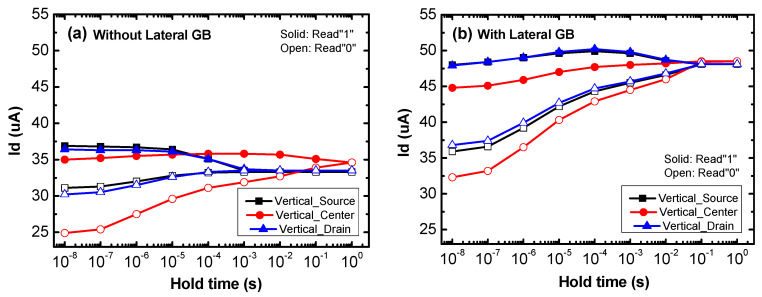
The retention characteristics of a poly-Si 1T-DRAM (**a**) without a lateral GB and (**b**) with a lateral GB according to a vertical GB’s location.

Figure 6a,b show the retention characteristics of poly-Si 1T-DRAM devices with varied vertical GB locations. The simulated devices of Figure 6a have no lateral GB and have only a single vertical GB in the source, center and drain locations, while the devices of Figure 6b have a lateral GB at 12 nm with a vertical GB that is also in the source, center and drain locations. As shown in Figure 6a, the retention characteristics are different depending on the vertical GB location. In Vertical_Source and Vertical_Drain, the Read “0” current hardly changes and the Read “1” current decreases with hold time, while in Vertical_Center, the Read “0” current increases with the hold time and Read “1” current is almost constant. However, Figure 6b shows that the devices with a lateral GB have relatively similar retention characteristics regardless of the vertical GB location. In all devices with a lateral GB, the change in the Read “0” current is significantly larger than the change in the Read “1” current. This demonstrates that the transient characteristics of a memory device with a lateral GB are hardly affected by its vertical GB location.

**Figure 7:**
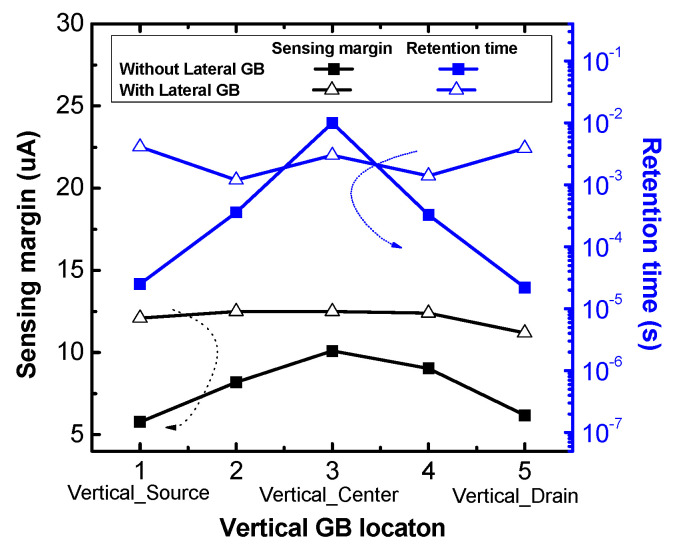
The sensing margin and retention time of poly-Si 1T-DRAM devices without and with a lateral GB according to a vertical GB location. Numbers from 1 to 5 on the *x*-axis are in order starting from the source region. The location numbers 1, 3 and 5 indicate Vertical_Source, Vertical_Center and Vertical_Drain, respectively.

Figure 7 shows the sensing margin and retention time of poly-Si 1T-DRAM devices without and with a lateral GB. The *x*-axis is the vertical GB location numbered in proportion to the distance from the source region. As shown in Figure 7, the devices without a lateral GB have a large change in sensing margin and retention time depending on the location of the vertical GB, while the devices with a lateral GB are influenced very slightly. Both the sensing margin and retention time of the devices without a lateral GB are degraded as a single vertical GB gets closer to the source or drain region. Also, in all vertical GB locations, the sensing margin of the devices with a lateral GB is larger than that of the devices without a lateral GB; the retention time is also greater with the lateral GB than without it except for Vertical_Center.

**Figure 8:**
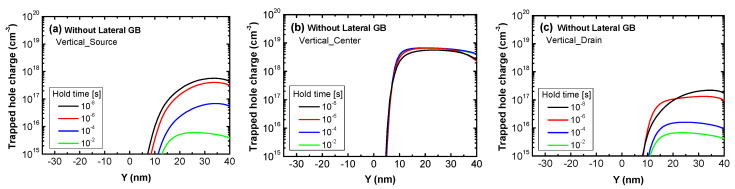
The trapped hole charge in the Read “1” operation of poly-Si 1T-DRAM devices that have no lateral GB as a function of a vertical GB’s position. The lines in each figure represent varied hold times to show their effect on the trapped hole charge. The devices are (**a**) Vertical_Source, (**b**) Vertical_Center and (**c**) Vertical_Drain.

We analyzed the trapped charge density to determine the change in retention characteristics with and without the lateral GB. Figure 8a–c show the trapped hole charge for the three vertical GB positions in devices without a lateral GB. The figures also show the change in trapped hole charge for varied hold times. In the figures, the *x*-axis is the position within a vertical GB and 0 nm and 40 nm indicate SiO_2_/Poly-Si interface and poly-Si body/buried oxide interfaces, respectively. The *y*-axis is the trapped hole charge in the read operation when operating in the order of Write “1”-Hold-Read“1.” Comparing Figure 8a–c, Vertical_Center has more trapped hole charge at 10 ns hold time than Vertical_Source and Vertical_Drain; the trapped hole charge of Vertical_Source and Vertical_Drain rapidly decreases with hold time while that of Vertical_Center hardly changes. This is because the vertical GB in Vertical_Source or Vertical_Drain is close to an n-type region, making it difficult for holes to be trapped in the GB and large numbers of holes are recombined with electrons. Thus, without lateral GB devices, the Read “1” current of both devices decreases faster with the hold time while the Vertical_Center’s Read “1” current is almost constant over time.

**Figure 9:**
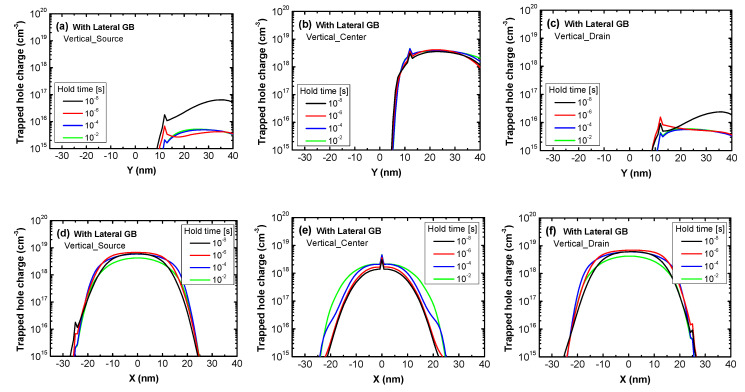
The trapped hole charge of poly-Si 1T-DRAM devices with lateral GB in the Read “1” operation as a function of the position within (**a**–**c**) a vertical GB and (**d**–**f**) a lateral GB. The lines in each figure represent varied hold times to show their effect on the trapped hole charge. The devices are (**a**) and (**d**) Vertical_Source; (**b**) and (**e**) Vertical_Center; and (**c**) and (**f**) Vertical_Drain.

Figure 9a–f show the change of trapped hole charge in poly-Si 1T-DRAM devices with a lateral GB depending on different vertical GB positions over varied hold times. Figure 9a–c and Figure 9d–f represent the trapped hole charge according to the position within a vertical GB and lateral GB, respectively. In other words, the *x*-axis of Figure 9a–c represents Y, the vertical location in the poly-Si body and the *x*-axis of Figure 9d–f represents X, the lateral location in the body. For the simulated devices with a gate length of 70 nm, the gate is from −35 nm to 35 nm in the X direction. As shown in Figure 9a–c, each device with different vertical GB locations has a trapped hole charge peak at 12 nm due to the lateral GB. Vertical_Source and Vertical_Drain in Figure 9a,c have less trapped hole charge and a larger time-dependent reduction of trapped hole charge than Vertical_Center in Figure 9b because their vertical GB is located near the n-type source or drain region where there are many electrons. However, Figure 9d–f indicate that the hole charge trapped within the lateral GB changes little over varied hold times regardless of the vertical GB location. In the figures, the trapped hole charge has a peak at each vertical GB location and more hole charges can be trapped in the lateral GB than in the vertical GB because the lateral GB’s region is wider; the gate length of the devices is 70 nm while their body thickness is 40 nm. Therefore, the hole charges trapped in the lateral GB have a dominant effect on the Read “1” current of the devices with a lateral GB and since the trapped charge has a similar value regardless of the vertical GB location, the Read “1” current of the three devices in Figure 6b also changes over time with a similar trend.

**Figure 10:**
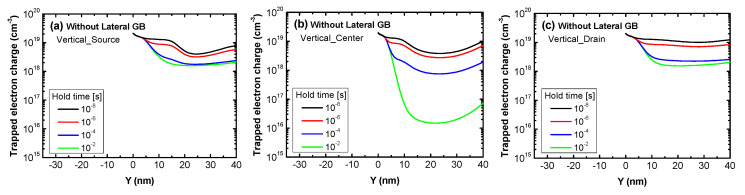
The trapped electron charge in the Read “0” operation of poly-Si 1T-DRAM devices that have no lateral GB as a function of the position within a vertical GB. The lines in each figure represent varied hold times to show their effect on the trapped electron charge. The devices are (**a**) Vertical_Source, (**b**) Vertical_Center and (**c**) Vertical_Drain.

Similar to the trapped hole charge, the change in the Read “0” current with hold time in Figure 6a,b can be analyzed using the change in trapped electron charge. Figure 10a–c show the change in trapped electron charge with hold time during the Read “0” operation in the three devices without a lateral GB and with varied vertical GB locations. The trapped charge decreases with hold time due to electron de-trapping and in contrast to the trapped hole charge during the Read “1” operation, the change of the trapped electron charge in Vertical_Source and Vertical_Drain with hold time in the Read “0” operation is smaller than that of Vertical_Center. Since a vertical GB is located near the source or drain, electron de-trapping is difficult due to the electrons supplied from the electron-rich n-type source or drain, even for long hold times. This affects the Read “0” current, thus the Read “0” current of Vertical_Source and Vertical_Drain, which has a smaller trapped electron charge change with hold time, hardly changes compared to Vertical_Center as seen in Figure 6a.

**Figure 11:**
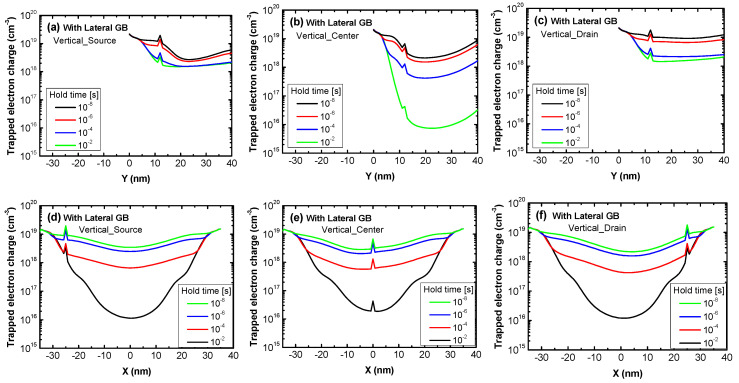
The trapped electron charge of poly-Si 1T-DRAM devices having a lateral GB in the Read “0” operation as a function of the position within (**a**–**c**) a vertical GB and (**d**–**f**) a lateral GB. The lines in each figure represent varied hold times to show their effect on the trapped electron charge. The devices are (**a**) and (**d**) Vertical_Source; (**b**) and (**e**) Vertical_Center; and (**c**) and (**f**) Vertical_Drain.

Figure 11a–f show the trapped electron charge of Vertical_Source, Vertical_Center and Vertical_Drain with a lateral GB within a vertical and lateral GB according to hold time. Since devices with a lateral GB have one lateral GB and one vertical GB in their body, trapped electron charge as a function of the position within the GB in each direction was analyzed. In Figure 11a–c, all devices have a trapped charge peak at their 12 nm lateral GB location and the trapped electron charge of Vertical_Source and Vertical_Drain in the Read “0” operation has a smaller change than that of the Vertical_Center for the same reason as the devices without a lateral GB. Figure 11d–f indicate that electrons trapped in the lateral GB also decrease with the hold time due to electron de-trapping and peak at the vertical GB location. However, the amount of time-dependent reduction of electrons trapped in the lateral GB is similar in all devices regardless of the vertical GB location because they share the same lateral GB location. More electrons are trapped in the lateral GB’s region which is wider than that of the vertical GB, so trapped electron charge in the lateral GB has a greater influence on the Read “0” current of the lateral GB devices. As a result, the Read “0” current of Vertical_Source, Vertical_Center and Vertical_Drain have almost the same increments with hold time due to their similar decreasing trapped electron charge trends.

Trapped charges can also be used to analyze the sensing margin and retention time of poly-Si 1T-DRAM according to the presence of a lateral GB. By comparing Figure 10a–c and Figure 11a–f, it can be seen that the total trapped electron charge of the lateral GB devices is larger than that of devices without a lateral GB due to the wider GB region. This lowers the Read “0” current of the lateral GB device when the hold time is small, resulting in a large difference from the Read “1” current. Conversely, the Read “0” current of devices without a lateral GB with their relatively smaller trapped electron charge does not decrease significantly compared with lateral GB devices at a hold time of 10 ns. And in particular, as can be seen in Figure 10a,c, Vertical_Source and Vertical_Drain without a lateral GB have little change in their trapped electron charge with hold time from the positional effect of the vertical GB, thus the Read “0” current hardly changes with the hold time. This means the Read “0” current of devices without a lateral GB is not much lower than the Read “1” current even for short hold times, causing the sensing margin to deteriorate. Therefore, lateral GB devices with more electron charge trapped in the GB have a larger sensing margin than those without a lateral GB.

Also, as shown in Figure 7, retention time is almost constant in lateral GB devices regardless of the vertical GB location, while in devices without a lateral GB, the retention time decreases as the vertical GB is located closer to the source or drain region. In these devices, as the vertical GB position approaches the source or drain region, the trapped hole charges are easily de-trapped due to recombination with electrons supplied from those regions. Retention time also tends to be larger in lateral GB devices than those without a lateral GB except for Vertical_Center which has the smallest difference in sensing margin due to its lateral GB. The Vertical_Center device with a lateral GB has a larger sensing margin than Vertical_Center without a lateral GB but its retention time is smaller because the device with a lateral GB has more hold time-dependent increments of Read “0” current. As shown in Figure 6a,b, Vertical_Center operates as a memory device while accommodating the change of the Read “0” current with hold time in devices with or without a lateral GB.

**Figure 12:**
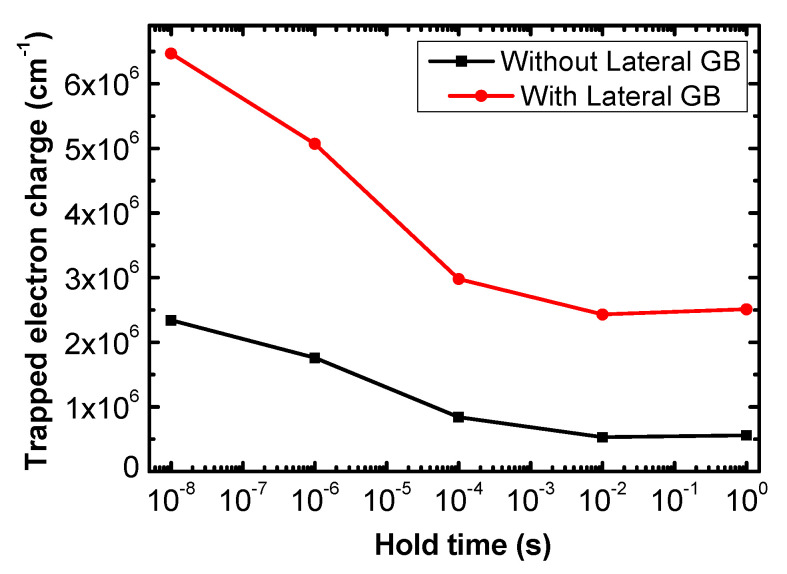
The change of the trapped electron charge of Vertical_Center without and with lateral GB according to the hold time during the Read “0” operation.

Figure 12 shows the change of the total trapped electron charge density of Vertical_Center devices with hold time during the Read “0” operation. In lateral GB devices, electron de-trapping occurs in both vertical and lateral GBs, so electrons decrease with a larger rate of change compared to devices without a lateral GB. Thus, the “0” current of lateral GB devices increases with hold time, reducing the retention time.

## 4. Conclusions

In this paper, we evaluated the effect of a single lateral GB’s location on a poly-Si 1T-DRAM’s memory performance. The sensing margin and retention time as a function of the lateral GB location have a cross point. The devices with a lateral GB near the cross point have trade-off trends between sensing margin and retention time and as the lateral GB location moves further away from the cross point, the memory performance of the devices deteriorates. For devices with a lateral GB near the cross point, the sensing margin decreases and retention time increases with the GB location because the trapped hole charge increases while the electron charge decreases. The reduction of trapped electron charge deteriorates the sensing margin while the increment of trapped hole charge temporarily increases retention time due to slower hole de-trapping.

We also studied the effect of a lateral GB by comparing the memory performance of devices without and with a lateral GB. In lateral GB devices, the lateral GB’s trapped charge is larger than that of the vertical GB, thus retention characteristics hardly change with the vertical GB location. On the other hand, in devices without a lateral GB, the difference in trapped charge with the vertical GB location causes differences in memory performance. In devices without a lateral GB and with a vertical GB near the n-type source or drain region, hole de-trapping easily occurs while electron de-trapping hardly occurs due to the electrons supplied by the source or drain. This deteriorates memory performance and increases its variation with the vertical GB location.

In summary, a lateral GB in a poly-Si 1T-DRAM device reduces variation in memory performance caused by the location of the vertical GB which is randomly determined during the fabrication, so devices with a lateral GB can operate stably as a memory device regardless of the vertical GB location. Therefore, the presence of a lateral GB should be considered in the poly-Si 1T-DRAM device fabrication process.

## Figures and Tables

**Table 1:** Device parameters for poly-Si 1T DRAM simulation.

Parameter	Value
Gate Length (L_g_)	70 nm
Body Thickness (T_body_)	40 nm
Buried Oxide Thickness (T_box_)	100 nm
Gate Oxide Thickness (T_ox_)	40 Å
Source/Drain doping concentration (Arsenic)	$1\times 10^{20}{\mathrm{cm}}^{-3}$
Channel doping concentration (Boron)	$1\times 10^{18}{\mathrm{cm}}^{-3}$
Substrate doping concentration (Boron)	$1\times 10^{16}{\mathrm{cm}}^{-3}$

**Table 2:** Bias and time conditions for the transient operation of poly-Si 1T-DRAM cells.

Operation	Write “1”	Write “0”	Read	Hold
V_g_ (V)	−2.0	0	1.2	0
V_d_ (V)	1.0	−1.5	0.1	0
Time (ns)	500	150	10	-
